# Cedrol ameliorates inflammatory bowel disease via mitochondrial biogenesis, gut microbiota restoration, and intestinal barrier repair

**DOI:** 10.3389/fphar.2025.1619537

**Published:** 2026-01-02

**Authors:** Mo-Rong Xu, Chia-Hsin Lin, Chung-Hsuan Wang, Sheng-Yang Wang

**Affiliations:** 1 Doctoral Program in Microbial Genomics, National Chung Hsing University and Academia Sinica, Taichung, Taiwan; 2 Department of Forestry, National Chung-Hsing University, Taichung, Taiwan; 3 Special Crop and Metabolome Discipline Cluster, Academy Circle Economy, National Chung Hsing University, Taichung, Taiwan; 4 Agricultural Biotechnology Research Center, Academia Sinica, Taipei, Taiwan

**Keywords:** inflammatory bowel disease (IBD), intestinal bacteria, mitochondrial biogenesis, cedrol, ATP, SCFA

## Abstract

**Introduction:**

Inflammatory bowel disease (IBD) is a chronic inflammatory disorder of the gastrointestinal tract, primarily characterized by impaired intestinal barrier function. Enhancing mitochondrial ATP biosynthesis and restoring gut microbiota and metabolic balance may contribute to intestinal barrier repair and serve as a therapeutic strategy for IBD. Cedrol, the major component of *Cunninghamia lanceolata* var. *konishii* wood essential oil, is known for its anti-inflammatory and anti-bacterial properties. However, its effects on gut health remain unclear.

**Methods:**

To evaluate the therapeutic effects of cedrol on inflammatory bowel disease (IBD), we assessed the expression of mitochondrial biogenesis and tight junction proteins using Western blotting. Additionally, we analyzed changes in gut microbiota and their metabolites, as well as colonic metabolites, through 16S rRNA gene sequencing, GC-MS, and ^1^H NMR analysis.

**Results:**

This study investigated the therapeutic potential of cedrol in an IBD model and found that it promotes colonic repair and enhances mitochondrial function by increasing ATP production and upregulating key proteins (SIRT-1, PGC-1α, Nrf2, *p*-AMPK/AMPK, TFAM). Furthermore, cedrol modulates gut microbiota balance by reducing *Anaerostipes hadrus* and *Turicibacter sanguinis* while increasing *Blautia glucerasea* and *Anaerotaenia torta*. It also elevates SCFA levels, supporting metabolic balance and the expression of tight junction protein expression, thereby strengthening the intestinal barrier and alleviating colitis.

**Conclusion:**

In conclusion, cedrol effectively restores mitochondrial function, modulates gut microbiota and metabolic balances, and enhances intestinal barrier integrity, highlighting its potential as a therapeutic candidate for IBD.

## 1 Introduction

Inflammatory bowel disease (IBD) is a chronic inflammatory disorder of the gastrointestinal tract with a steadily increasing incidence, particularly in developing countries. This rise imposes substantial medical, economic, and quality-of-life burdens, making IBD a significant global public health concern (Dharni et al., 2024). Despite extensive research, its precise etiology remains unclear. Current hypotheses suggest that IBD pathogenesis results from complex interactions among environmental factors, gut microbiota, and genetic predisposition, ultimately leading to intestinal barrier dysfunction, tissue damage, and immune dysregulation (Glassner et al., 2020). The unknown etiology not only limits treatment options but also increases the risk of disease-related complications, including a heightened susceptibility to colorectal cancer (CRC) (Shah and Itzkowitz, 2022). Therefore, advancing disease prevention strategies to facilitate early diagnosis and developing innovative therapeutic approaches are crucial to mitigating IBD.

Emerging evidence further indicates that IBD pathogenesis is closely linked to tight junction disruption in intestinal epithelial cells, mitochondrial dysfunction, gut microbiota dysbiosis, and metabolic disorders (Lin et al., 2018; Sun et al., 2024). The hallmark of IBD is intestinal epithelial damage and compromised gut barrier function. When the intestinal barrier is disrupted, pathogens and toxins infiltrate the intestinal lumen, triggering inflammatory responses that drive disease onset and progression. Clinically, this manifests as abdominal pain, diarrhea, hematochezia, and weight loss. Notably, disruption of epithelial integrity is considered an early pathogenic event in IBD, with alterations in tight junction protein expression identified as a key molecular mechanism underlying increased gut permeability and loss of barrier integrity (Hyun, 2021). Thus, restoring intestinal barrier function represents a promising strategy for treating and preventing IBD.

Mitochondrial dysfunction is also implicated in IBD pathogenesis (Roediger, 1980; Mancini et al., 2020). Since intestinal epithelial cells are highly enriched in mitochondria, mitochondrial impairment compromises ATP production, affecting cellular self-renewal, differentiation, and overall intestinal homeostasis (Jackson and Theiss, 2020). Moreover, the integrity of tight junctions is energy-dependent, further understanding the critical role of mitochondrial function in maintaining gut barrier integrity (Van Der Flier and Clevers, 2009). Previous studies suggest that improving mitochondrial quality may benefit cells or animals with mitochondrial dysfunction (Russell et al., 2020). Through fusion and fission processes, mitochondria can be remodeled to stabilize intracellular ATP, ROS, and mtDNA levels (Mancini et al., 2020). Importantly, mitochondrial biogenesis provides a mechanism for restoring mitochondrial pool and function, thereby supporting epithelial energy demands and promoting mucosal healing, highlighting its therapeutic significance for IBD.

Additionally, gut microbiota dysbiosis plays a crucial role in IBD pathogenesis (Cremon et al., 2018). The gut microbiota is essential for various physiological processes, including intestinal cell proliferation and differentiation, energy metabolism, immune system modulation, and pathogen defense. Beneficial bacteria help prevent pathogenic overgrowth, enhance gut barrier function, and regulate immune responses, thereby mitigating disease progression (Cremon et al., 2018). Microbial metabolites, particularly short-chain fatty acids (SCFAs) and bile acids, significantly influence intestinal barrier integrity and systemic immunity (Liu et al., 2022). These metabolites regulate mucosal immunity, angiogenesis, epithelial renewal, and metabolic homeostasis. IBD is characterized by dysregulated metabolism of SCFAs, bile acids, tryptophan, and amino acids, leading to altered host-microbe interactions and exacerbating intestinal barrier dysfunction and chronic inflammation (Zhou et al., 2023). Therefore, restoring gut microbiota composition and metabolic balance represents a promising therapeutic strategy for IBD.

*Cunninghamia lanceolata* (Lamb.) Hook. var. *konishii* [Cupressaceae] is an endemic tree species in Taiwan, with cedrol as the primary component in its wood essential oil (Cheng et al., 2011). Cedrol, a sesquiterpenoid, exhibits various properties, including anti-inflammatory, analgesic, anticancer, antibacterial, and hair growth-promoting properties (Cheng et al., 2011; Chang et al., 2020; Zhang et al., 2021). Previous studies have shown that cedrol enhances ATP production in Caco-2 cells and increases tight junction proteins (Xu et al., 2025). However, the protective effects of cedrol on gut health in IBD remain unexplored.

In summary, restoring mitochondrial function, gut microbiota balance, and metabolic homeostasis may contribute to intestinal barrier repair. However, few studies have explored the mechanisms underlying cedrol’s potential effects on IBD remain unknown. This study aims to investigate the impact of cedrol on mitochondrial function, gut microbiota, metabolic balance, and intestinal barrier integrity using a dextran sulfate sodium (DSS)-induced mouse model of IBD. By elucidating the interplay among these four factors, this study seeks to provide a novel therapeutic strategy focused on intestinal barrier restoration, distinct from traditional anti-inflammatory approaches, ultimately contributing to developing innovative treatment strategies for IBD.

## 2 Materials and methods

### 2.1 Reagents and materials

Protease Inhibitor Cocktail (HC100-007, Goalbio, Taiwan), Phosphatase Inhibitor Cocktail (HC100-008, Goalbio, Taiwan), bovine serum albumin (Gibco, United States), BioRad protein assay dye reagent concentrate (BioRad, United States), 5 X Protein Sample Dye (GM47-b, GeneMark, Taiwan), PVDF transfer membrane (Revvity, United States), EZblocker (GenePure, Taiwan), WesternBrightTM ECL (Advansta, United States), 10% Neutral Phosphate Buffered Formalin (Leica Surgipath, United States), T-PERTM Tissue Protein Extraction Reagent (Thermo Fisher Scientific, United States), Mouse IL-6 Valuvine™ ELISA (VAL604G, Novus Biologicals, China), Mouse IL-β Valuvine™ ELISA (VAL601, Novus Biologicals, China), Mouse TNF-α Valuvine™ ELISA (VAL609, Novus Biologicals, China), Deproteinizing Sample Preparation Kit - TCA (ab204708, Abcam, UK), ATP Assay Kit (Colorimetric/Fluorometric) (ab83355, Abcam, UK), CheKine™ Micro Lipid Peroxidation (MDA) Assay kit (KTB1050-EN, Abbkine, United States), RNeasy Mini Kit (QIAGEN, Germany), DSS (MW 40,000, Thermo Scientific™, United States), Methanol-d4 (151947-10G-GL, Sigma-Aldrich, Germany), Deuterium oxide (151882-100G, Sigma-Aldrich, Germany), 3-(Trimethylsilyl)propionic-2,2,3,3-d4 acid sodium salt (TSP) (269913-1G, Sigma-Aldrich, Germany).

### 2.2 Extraction of cedrol

The *C. lanceolata* var. *konishii* wood was generously provided by Dr. Min Jay Chung of the Experimental Forest Management Office, Taiwan. Essential oil was initially extracted from the wood through steam distillation. Following extraction, the essential oil underwent recrystallization to obtain high-purity cedrol. The preparation of cedrol followed established methods, with its purity and structure confirmed through GC-MS and NMR spectroscopy (Xu et al., 2025).

### 2.3 Animals

All animal procedures adhered to the guidelines set forth by the Institutional Animal Care and Use Committee (IACUC) of National Chung Hsing University (IACUC No. 112-065). The mice in this experiment were from BioLASCO (Yilan, Taiwan) and used 7-week-old female C57BL/6 mice. Mice were group-housed under controlled conditions, including a temperature of 22United States°C ± 2 °C, relative humidity of 50%–60%, and a 12-h light/dark cycle, with *ad libitum* access to water and food. After a 1-week acclimatization period, the animals were used for experimental procedures.

### 2.4 Experimental design for DSS-induced colitis in mice

To establish an inflammatory bowel disease (IBD) model, dextran sulfate sodium (DSS, MW 40,000, Thermo Scientific™, United States) was used in mice, following established protocols (Liu et al., 2017). C57BL/6 female mice (7-week-old) underwent a 1-week acclimation period and were randomly assigned into three groups (n = 6 per group). The experiment was conducted in two phases (Supplementary Figure S1), with 18 mice in each phase (36 mice in total). Based on previous studies, this two-phase design included an acute induction phase and a recovery phase, allowing assessment of both anti-inflammatory effects and the drug’s ability to sustain remission and promote mucosal healing, as inflammation and tissue abnormalities can persist beyond apparent clinical recovery (Gelmez et al., 2022; Jeong et al., 2025).

#### 2.4.1 Phase 1: effects of cedrol on DSS-induced colitis

In this phase, the effects of cedrol on DSS-induced colitis were evaluated. The cedrol gavage dose (100 mg/kg/day) was chosen based on previous studies in mice, balancing efficacy and safety (Chen et al., 2020; Chien et al., 2022; Kang et al., 2025). The control group received regular drinking water and was gavaged with corn oil for 7 days. DSS and DSS + cedrol groups were given 2.5% (w/v) DSS in distilled water to induce acute colitis. The DSS group was gavaged with corn oil, while the DSS + cedrol group received 100 mg/kg/day of cedrol dissolved in corn oil by gavage for 7 days. On the seventh day, the mice were sacrificed, and various tissues, including the colon and rectum, were carefully harvested. These tissues were then processed and preserved appropriately for subsequent histological and biochemical analyses to evaluate the experimental outcomes in detail.

#### 2.4.2 Phase 2: effects of cedrol on colitis recovery

The second phase examined cedrol’s effects during the recovery period following DSS-induced colitis. The control group continued to receive normal drinking water and corn oil gavage for 13 days. In the DSS and DSS + cedrol groups, 2.5% (w/v) of DSS in distilled water was administered for 6 days to induce colitis, after which it was replaced with distilled water. The DSS group continued to be gavaged with corn oil, while the DSS + cedrol group received 100 mg/kg/day of cedrol for 13 days. On the thirteenth day, mice were sacrificed.

### 2.5 Disease activity index (DAI)

The severity of colitis in the model was assessed using the Disease Activity Index (DAI), which considers body weight loss, stool consistency, and the presence of blood in feces (Shen et al., 2022). The DAI score was calculated: DAI = Combined score of (weight loss + stool consistency + fecal blood).

### 2.6 Histological analysis (H&E staining analysis)

The severity of inflammation in the acute colitis model was assessed using hematoxylin and eosin (H&E) staining. Colon tissues were first collected and fixed in 10% neutral buffered formalin, followed by dehydration. The dehydrated samples were embedded in paraffin and sectioned into 3 μm slices. Finally, the slides were stained with H&E to evaluate histological damage (Shen et al., 2022). The histological grading of intestinal inflammation was defined by the infiltration of immunocytes and the degree of epithelial/crypt damage.

### 2.7 Enzyme-linked immunosorbent assay (ELISA) detection

Serum levels of pro-inflammatory cytokines in colitis-induced mice were assessed using ELISA. Blood was collected from the heart into serum micro-collection tubes (365967, Becton Dickinson, United States) and left at room temperature for 1 h. Afterward, samples were centrifuged at 1,600 g for 10 min at room temperature, and the serum was stored at −80 °C. TNF-α, IL-1β, and IL-6 levels were performed by mouse ELISA kit (Novus Biologicals, China) and according to the manufacturer’s instructions.

### 2.8 ATP assay

The ATP content in DSS-induced colitis mice treated with cedrol was evaluated using an ATP assay. ATP extraction followed the instructions of the ATP Assay Kit (Colorimetric/Fluorometric) (ab83355, Abcam, UK), with modifications based on previous studies (Spoorthi et al., 2020). To begin, 20 mg of colon tissue was washed with cold PBS, homogenized in 200 μL of ATP Assay Buffer, and centrifuged at 13,000 g for 5 min at 4 °C to remove insoluble materials. The supernatant was collected, and protein content was quantified using the BCA method, adjusting concentrations to a uniform level (800 μg) with ATP Assay Buffer. Following the Deproteinizing Sample Preparation Kit-TCA (ab204708, Abcam, UK) instructions, deproteinization was then performed to eliminate interfering enzymes. Reaction reagents were added as directed, and the mixture was incubated in the dark for 30 min. Absorbance was measured at 570 nm using a Multilabel Microplate Reader (Hidex, Finland). The absorbance value for each well was adjusted by subtracting the blank and sample background absorbance values (OD Reaction - OD Background Reaction). A standard curve was plotted with ATP standard concentrations and their corresponding absorbance values, allowing for the calculation of sample ATP concentrations based on the standard curve formula.

### 2.9 MDA assay

Malondialdehyde (MDA) is a widely recognized biochemical marker for oxidative stress. Colon tissue samples (20 mg each) were collected, and MDA levels were measured following the protocol of the CheKine™ Micro Lipid Peroxidation (MDA) Assay Kit (KTB1050-EN, Abbkine, United States). Absorbance values were read at 532 nm and 600 nm. The corrected absorbance for each well was calculated by subtracting the average blank OD value (OD Reaction - OD Background Reaction). MDA levels were then determined using the formula below, where *A* represents the corrected absorbance: MDA levels (nmol/mg protein) = *A* × 51.6 / protein concentration (mg/mL).

### 2.10 Evaluation of mtDNA gene expression via quantitative PCR (qPCR)

The expression of mtDNA genes in DSS-induced colitis mice treated with cedrol was evaluated using quantitative PCR (qPCR). Colonic tissues were collected from all mice (n = 6 per group for Control, DSS, and DSS + cedrol) on day 7 after treatment. Each mouse was analyzed individually. RNA was extracted following the RNeasy Mini Kit protocol (QIAGEN, Germany), and the extracted RNA was stored at −80 °C. SuperSAMScript IV Reverse Transcriptase (GRT004L, GeneMark, Taiwan) reverse-transcribes total RNA into cDNA. A template of 200 ng RNA was used for cDNA synthesis, conducted with a PCR Thermal Cycler (PC-818A, Astec, Japan) and stored at −20 °C. The qPCR analysis targeted mtDNA and β-actin genes (endogenous control), with primers designed as follows:

mtDNA: Forward: 5′-TGA​ACG​GCT​AAA​CGA​GGG​TC-3′, Reverse: 5′-AGC​TCC​ATA​GGG​TCT​TCT​CGT-3’
β-actin: Forward: 5′-TCA​GCA​AGC​AGG​AGT​ACG​ATG-3′, Reverse: 5′-AAC​GCA​GCT​CAG​TAA​CAG​TCC-3’

The qPCR reaction used PowerUp™ SYBR™ Green Master Mix (Thermo Fisher Scientific, United States) and was performed on a StepOne™ Real-Time PCR System (Applied Biosystems, United States). Data were analyzed using StepOne Software v2.3 (Thermo Fisher Scientific, United States), with β-actin as the internal control. Relative gene expression was calculated using the 2^^−ΔΔCt^ method.

### 2.11 Evaluation of mitochondrial biogenesis and tight junction protein expression by Western blotting

The expression of mitochondrial biogenesis and tight junction proteins in the intestinal tissues of mice was analyzed using Western blotting. Colon tissue samples (25 mg) were homogenized in 400 μL of lysis buffer containing T-PER™ Tissue Protein Extraction Reagent (Thermo Fisher Scientific, United States), supplemented with 1% protease inhibitor cocktail (HC100-007, Goalbio, Taiwan) and phosphatase inhibitor cocktail (HC100-008, Goalbio, Taiwan). The samples were sonicated on ice at 60% intensity for 10 s, repeated five times, using an Ultrasonic Processor (UP-800, ChromTech, United States), and then placed on ice for 10 min to ensure complete cell lysis. The supernatant (proteins) was collected after centrifugation at 16,000 g for 5 min at 4 °C and stored at −80 °C. Protein concentration was standardized to 90 μg/mL using the BCA method, and proteins were denatured at 50 °C at 1,500 rpm for 5 min. Proteins were separated via electrophoresis and transferred to a PVDF membrane (Revvity, United States) at 300 mA for 1-2 h. Following the transfer, the membrane was washed three times with TBST buffer and blocked with protein-free blocking solution (EZblocker, GenePure, Taiwan).

The membrane with primary antibodies targeting different proteins: occludin (1:10000, 27260-1-AP, Proteintech, China), TFAM (1:20000, 22586-1-AP, Proteintech, China), PGC-1α (1:10000, 66369-1-lg, Proteintech, China), claudin-1 (1:500, 51-9000, Invitrogen, United States), claudin-3 (1:1,000, 34-1700, Invitrogen, United States), AMPKα1 (1:1,000, 07-350, Millipore Sigma, United States), p-AMPKα (1:1,000, 2535, Cell Signaling Technology, United States), SIRT-1 (1:1,000, 9475s, Cell Signaling Technology, United States), Nrf2 (1:1,000, 12721, Cell Signaling Technology, United States), and β-actin (1:5000, SC-47778, Santa Cruz, UK) were incubated overnight at 4 °C. After primary incubation, the membrane was washed with TBST buffer and incubated with either Goat anti-Mouse IgG (HRP) (1:10000, ARG65350, Arigo Biolaboratories, China) or Rabbit IgG (HRP) (1:10000, abs-22-200, Asia Bioscience, Taiwan) secondary antibodies at room temperature for 2 h with gentle shaking. Following a final TBST wash, the immune-reactive bands were detected using WesternBright™ ECL (Advansta, United States) and visualized with a chemiluminescence.

### 2.12 Immunohistochemistry (IHC) assessment of TOMM20 expression in mouse intestine

Colonic tissues were collected from all mice (n = 6 per group for Control, DSS, and DSS + cedrol) on day 7 after treatment, and each mouse was analyzed individually. These tissues were used for immunohistochemistry (IHC) to evaluate the expression of translocase of the outer mitochondrial membrane 20 (TOMM20) in the mouse intestine. Tissue samples were fixed in paraffin and sectioned to slices. Then, the sections were deparaffinized, and antigen retrieval was conducted using Antigen Retrieval Tris-EDTA HIER Solution (TES999, Scytek) for 30 min. Following antigen retrieval, sections were incubated with the TOMM20 antibody (1:100, ab186735, Abcam, UK) in the dark for 1 h. After washing with TBST, sections were further incubated with Alexa Fluor® 488 AffiniPure™ Goat Anti-Rabbit IgG (H + L) (1:100, 111-545-003, Jackson ImmunoResearch, United States) in the dark for 30 min. Then, the sections were washed with TBST and mounted using UltraCruz® Hard-set Mounting Medium containing DAPI (sc-359850, Santa Cruz, United States). TOMM20 expression was observed using a microscope (ECLIPSE Ci, Nikon, Japan) under a 20× objective lens, and images were captured via a microscope imaging system (SGHD-3.6, SAGE Vision, Taiwan) connected to SG Image V2.3 software (Taiwan). ImageJ software (National Institutes of Health, United States) was used for image processing, channel merging, and fluorescence quantification.

### 2.13 The effect of cedrol on gut bacteria in DSS-induced colitis using 16S rRNA gene sequencing

The effect of cedrol on intestinal bacteria in DSS-induced colitis mice was assessed through 16S rRNA gene sequencing. Cecal samples (100 mg) were collected, and DNA extraction was performed following the QIAamp Fast DNA Stool Mini Kit protocol (QIAGEN, Germany). The extracted DNA was stored at −20 °C, and its concentration was determined by spectrophotometry at 260 and 280 nm. Next, third-generation PacBio sequencing (BIOTOOLS, Taiwan) was conducted on the cecal microbiota. The 16S gene from each sample was amplified using universal 16S primers (27F + 1492R) through polymerase chain reaction (PCR). The labeled DNA was then quality-checked and prepared for SMRTbell library construction. Library preparation involved purification with AMPure PB beads, DNA repair, and adapter ligation, followed by PacBio sequencing. The DADA2 pipeline processes HiFi sequences and generates Amplicon sequence variants (ASVs), with steps including quality control, redundancy and chimera removal, and clustering. ASVs were compared with databases (NCBI, GreenGenes, SILVA, eHOMD, and UNITE) to obtain species identification and an abundance table (ASVs Table). The ASV table was then used for alpha diversity analysis, beta diversity analysis, taxonomic composition analysis, correlation analysis, and statistical testing.

### 2.14 Evaluation of cedrol’s effects on SCFAs in DSS-induced colitis by GC-MS

#### 2.14.1 Sample preparation

Short-chain fatty acids (SCFAs) in DSS-induced colitis mice were evaluated using gas chromatography-mass spectrometry (GC-MS). The SCFA quantification method was adapted from Bassett et al. (2019)(Bassett et al., 2019). Briefly, 200 mg of fecal samples were homogenized in 1 mL of 4 °C PBS, vortexed for 10 min, and centrifuged at 21,000 g for 10 min at 4 °C. Then, 500 µL of supernatant was added with 2 µL of two-ethylbutanoic acid as internal standard, 100 µL of 37% HCl, and 400 µL of n-hexane, vortexed, and centrifuged again. Next, 300 µL of supernatant was dried with 50 mg of Na_2_SO_4_, centrifuged. Then 160 µL supernatant was mixed with 40 μL MTBSTFA (N-tert-Butyldimethylsilyl-N-methyltrifluoroacetamide). The mixture was incubated at 80 °C for 1 h and 1 µL was injected into the GC-MS. All GC spectra were integrated using two-ethylbutanoic acid as an internal standard, and integration values for each group were normalized to the control group. SCFA standard spectra are shown in Supplementary Figure S2.

#### 2.14.2 GC-MS conditions and quantification

GC-MS analysis was conducted using an ITQ 900 mass spectrometer (Thermo Fisher Scientific, United States) and the system with a DB-5MS column (30 m × 0.25 mm × 0.25 μm, Agilent, United States). Helium served as the carrier gas (1 mL/min, split ratio 10:1). The injector and detector were set at 270 °C and 250 °C, respectively. The column temperature started at 50 °C, ramped to 80 °C (15 °C/min), then to 105 °C (1 °C/min), followed by 300 °C (45 °C/min), held for 5 min. Compounds were identified via the Wiley/NBS and NIST databases and quantified using n-alkanes (C9–C24).

### 2.15 Metabolomic analysis of colon tissue using ^1^H NMR

#### 2.15.1 Sample preparation for ^1^H NMR analysis

The ^1^H NMR analysis was adapted from previous study (Xu et al., 2025). Initially, 15 mg of colon tissue was extracted with 500 μL of ice-cold 70% methanol (MeOH) and processed using a tissue homogenizer (SH-100, KURABO, Japan) at 100 × 100 rpm for 30 s, repeated five times. The samples were vortexed on ice for 10 min to ensure homogenization of cellular metabolites. After centrifugation at 10,000 g for 15 min at 4 °C, the supernatant containing metabolites was transferred to a new 1.5 mL Eppendorf tube. The methanol was evaporated in a vacuum oven (Napco National, Saudi Arabia, Model 5831), leaving the tissue metabolites for analysis. The metabolic profile of the colon tissue extracts was analyzed using an NMR spectrometer, with a 1:1 mixture of deuterium oxide (D_2_O) and methanol-d_4_ (MeOD) as the solvent and 0.0002% TSP as the internal standard. For analysis, the dried metabolites were dissolved in 650 µL of the solvent mixture, of which 600 µL was transferred into a 5 mm NMR tube for ^1^H spectroscopy.

#### 2.15.2 ^1^H NMR data acquisition and analysis

All spectra were acquired on an Advance III-400 NMR spectrometer (Bruker, United States) at a proton frequency of 400 MHz and temperature of 300 K. MeOD served as an internal lock for cell extract samples, maintaining gradient shimming. The parameters for ^1H spectra were as follows: 90° pulse, 1,000 scans, 20 ppm spectral width, and a pre-scan delay of 6.5 s. Spectra were analyzed in TopSpin (version 3.5pl7, Bruker, Germany), with TSP used for zero chemical shift calibration and as an internal standard for integration and normalization. Integration and chemical shift data were exported to Excel, converted to CSV, compressed, and imported into MetaboAnalyst 6.0 for further analysis. Data normalization was performed through autoscaling. Initial analysis used unsupervised principal component analysis (PCA) to assess metabolic differences in colon tissue. Supervised sparse partial least squares discriminant analysis (sPLS-DA) was then applied to refine grouping and reduce environmental and systematic errors. sPLS-DA compared three groups: control, DSS, and DSS + cedrol, identifying significant metabolites from the loading plots of major predictors. Metabolites were identified using chemical shifts from the Human Metabolome Database (HMDB, http://www.hmdb.ca) and the Biological Magnetic Resonance Data Bank (BMRB, http://www.bmrb.wisc.edu) and cross-referenced with prior literature (Carneiro et al., 2019).

### 2.16 Statistical analysis

Data are presented as mean ± SD from at least three independent experiments. Statistical analysis was performed using GraphPad Prism 8.0 (Dotmatics, United States). One-way ANOVA followed by Tukey’s HSD *post hoc* test was applied, with *p* < 0.05 considered statistically significant.

## 3 Results

### 3.1 Cedrol attenuates symptoms of DSS-induced colitis in mice

**FIGURE 1:**
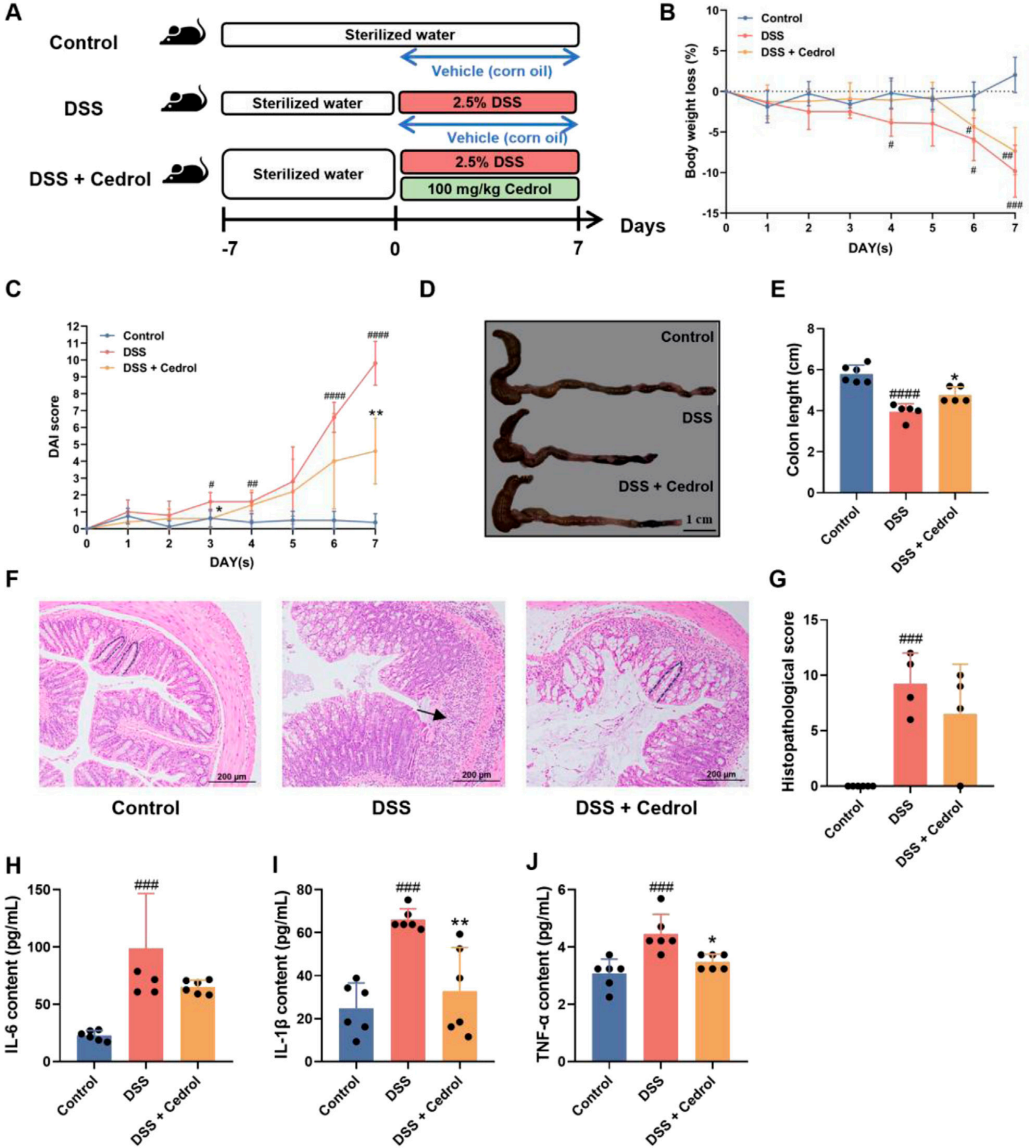
Cedrol mitigates the symptoms in DSS-induced colitis mice. **(A)** The design of the animal experiment. **(B)** The body weights of mice. **(C)** DAI scores. **(D)** Diarrhea scores. **(E)** Colon images and measurements of colon length were analyzed and compared across different experimental groups. **(F)** Representative histological H&E staining of sections of the colon (arrows indicate areas of massive inflammatory cell infiltration; dashed lines indicate crypt structures). **(G)** Histological scores of colonic tissue. **(H–J)** Serum levels of the IL-6, IL-1β, and TNF-α. The values are expressed as mean ± SD of more than three independent experiments. Statistical differences were analyzed with one-way ANOVA. # vs. control group (^#^ *p* < 0.05, ^##^ *p* < 0.01, ^###^ *p* < 0.001, ^####^ *p* < 0.0001) and * vs. DSS group (*p < 0.05, **p < 0.01, ****p < 0.0001). Control, corn oil; DSS, DSS-induced; DSS + Cedrol, DSS induced with cedrol treatment.

To evaluate the effects of cedrol on symptoms of DSS-induced colitis, mice in the control group received only corn oil, while induction groups were given either corn oil (DSS group) or 100 mg/kg of cedrol (DSS + cedrol group) via oral gavage. Induction groups drank 2.5% DSS for 7 days to establish acute colitis (Figure 1A). The results demonstrated a significant reduction in body weight loss in DSS-induced mice (Figure 1B). Disease Activity Index (DAI) scores revealed no colitis symptoms in the control group, whereas the DSS group have bloody stool and diarrhea symptoms starting on day 6, exhibited significantly higher scores. Cedrol treatment reduced bloody stool and significantly lowered DAI scores (Figure 1C). As shown in Figures 1D,E, the DSS group exhibited significantly short colon length compared to the control group, while cedrol treatment notably restored colon length. Histological analysis showed that the control group had intact colonic mucosa, while the DSS group exhibited crypt loss, inflammatory infiltration, and severe tissue damage. Cedrol treatment alleviated tissue inflammation and reduced histological scores compared to the DSS group (Figures 1F,G). These findings indicate that cedrol effectively mitigates DSS-induced mice symptoms.

The pro-inflammatory cytokines levels, as shown in Figure 1H–J, compared to the control group, DSS induction significantly increased TNF-α, IL-1β, and IL-6 levels. However, cedrol treatment significantly reduced IL-1β and TNF-α levels compared to the DSS group (Figures 1I,J). These results indicate that cedrol can decrease pro-inflammatory cytokines by DSS-induced and mitigate the inflammatory response through anti-inflammatory pathways.

### 3.2 Cedrol improves mitochondria function in DSS-colitis mice

**FIGURE 2:**
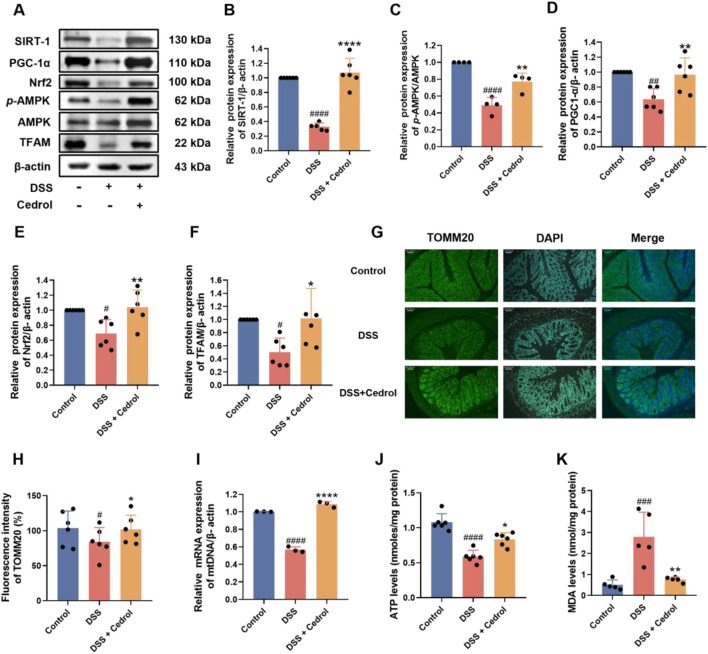
Cedrol enhances mitochondrial function in DSS-induced colitis. **(A)** Western blotting visual image of SIRT-1, PGC-1α, Nrf2, *p*-AMPK, AMPK, and TFAM. **(B–F)** Relative protein expression levels of SIRT-1, PGC-1α, Nrf2, TFAM, and p-AMPK/AMPK in the colon. **(G)** Fluorescence microscope images of colon tissues with TOMM20 antibody visualized at magnifications (×20). **(H)** The fluorescence intensity of TOMM20 under a fluorescence microscope and quantification results. **(I)** ATP levels. **(J)** Relative mRNA expression of mtDNA genes. **(K)** MDA levels. The values are expressed as mean ± SD of more than three independent experiments. Statistical differences were analyzed with one-way ANOVA. # vs. control group (^#^ *p* < 0.05, ^##^ *p* < 0.01, ^###^ *p* < 0.001, ^####^ *p* < 0.0001) and * vs. DSS group (*p < 0.05, **p < 0.01, ****p < 0.0001). Control, corn oil; DSS, DSS-induced; DSS + Cedrol, DSS induced with cedrol treatment.

This study evaluated the effects of cedrol on mitochondrial biogenesis protein expression in DSS-induced colitis mice using Western blotting. As shown in Figure 2A, DSS induction significantly reduced critical proteins in the mitochondrial biogenesis pathway, including SIRT-1 and *p*-AMPK/AMPK (Figures 2B,C), as well as PGC-1α and Nrf2 (Figures 2D,E). Consequently, the expression of the TFAM was also significantly decreased (Figure 2F). However, cedrol treatment effectively restored the expression of SIRT-1, *p*-AMPK/AMPK, PGC-1α, Nrf2, and TFAM expression levels. These results suggest that cedrol can reverse DSS-induced disruptions in mitochondria biogenesis and alleviate mitochondrial dysfunction by upregulating these critical proteins.

The effects of cedrol on the mitochondrial outer membrane protein TOMM20 in DSS-induced colitis mice were further examined using immunohistochemistry (IHC) of colon sections. The results showed that DSS induction significantly decreased the fluorescence intensity of TOMM20 compared to the control group. However, cedrol treatment significantly restored TOMM20 expression levels (Figures 2G,H). Additionally, DSS significantly decreased mtDNA gene expression relative to the control group, while cedrol treatment markedly upregulated mtDNA expression (Figure 2I). An ATP assay was conducted to assess ATP levels in colonic tissues, revealing that DSS significantly reduced ATP levels compared to the control, whereas cedrol treatment restored ATP levels (Figure 2J). Regarding oxidative stress, DSS induction significantly increased MDA levels, while cedrol treatment significantly reduced them (Figure 2K). These findings indicate that cedrol alleviates mitochondrial dysfunction by enhancing TOMM20 protein expression, promoting mtDNA gene and ATP production, and reducing oxidative stress.

### 3.3 Cedrol promotes recovery from DSS-induced colitis

**FIGURE 3:**
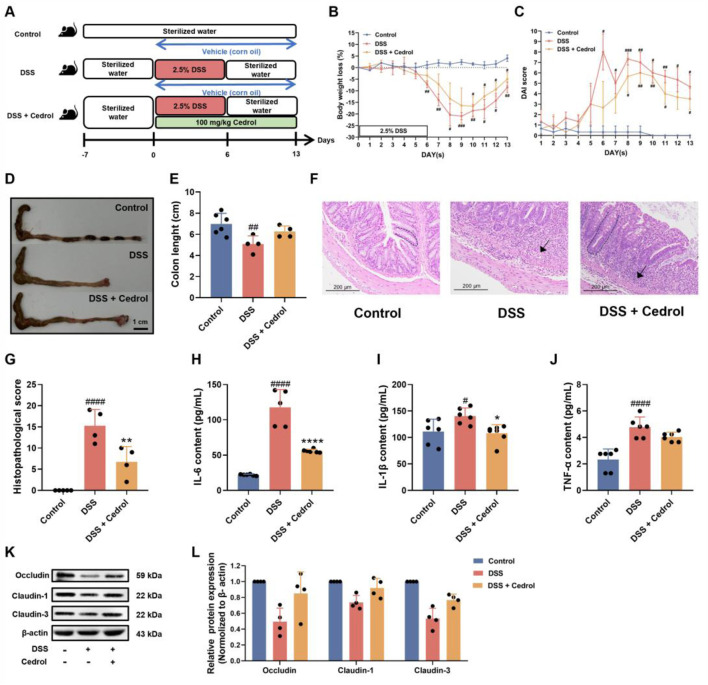
Cedrol promotes symptom recovery and intestinal mucosal barrier function in DSS-induced colitis. **(A)** The design of the animal experiment. **(B)** The body weights of mice. **(C)** DAI scores. **(D,E)** Colon images and measurements of colon length were analyzed and compared across different experimental groups. **(F)** Representative histological H&E staining of sections of the colon (arrows indicate areas of massive inflammatory cell infiltration; dashed lines indicate crypt structures). **(G)** Histological scores of colonic tissue. **(H–J)** Serum levels of the IL-6, IL-1β, and TNF-α. **(K)** Western blotting visual image of occludin, claudin-1, and claudin-3. **(L)** Relative protein expression levels of occludin, claudin-1, and claudin-3 in the colon. β-actin was used as the protein loading control. The values are expressed as mean ± SD of more than three independent experiments. Statistical differences were analyzed with one-way ANOVA. # vs. control group (^#^ *p* < 0.05, ^##^ *p* < 0.01, ^###^ *p* < 0.001, ^####^ *p* < 0.0001) and * vs. DSS group (*p < 0.05, **p < 0.01, ****p < 0.0001). Control, corn oil; DSS, DSS-induced; DSS + Cedrol, DSS induced with cedrol treatment.

To investigate cedrol’s impact on recovery from DSS-induced colitis in mice, we conducted an experimental study. The control group received corn oil, while two induction groups were administrated orally with either corn oil or 100 mg/kg of cedrol. Acute colitis was induced by providing 2.5% (w/v) DSS in drinking water for 6 days. After this, DSS was replaced with distilled water and sacrificed on day 13 (Figure 3A). Results indicated that body weight in the DSS group decreased significantly by day 6, reaching its lowest on day 9, confirming successful colitis induction. After day 9, weight loss stabilized with cedrol treatment, which mitigated the weight decline in the DSS group (Figure 3B). DAI scores further illustrated these effects: the control group showed no colitis symptoms, whereas the DSS group exhibited bloody stool and diarrhea from day 6, leading to significantly higher DAI scores compared to the control group (*p* < 0.05). Cedrol treatment reduced loose stool and lowered DAI scores (Figure 3C). Colon lengths highlighted additional improvement. The DSS group showed significantly shorter colon than controls, while the cedrol-treated group demonstrated extended colon length compared to the DSS group (Figures 3D,E). Histological analysis using H&E staining revealed that during recovery, cedrol-treated mice had reduced tissue inflammation and lower histopathological scores compared to the DSS group (Figures 3F,G). These findings demonstrated that cedrol can alleviate symptoms of DSS-induced colitis symptoms during the recovery.

To assess the influence of cedrol on pro-inflammatory cytokines in the recovery phase (Figures 3H–J). DSS induction markedly elevated cytokines (TNF-α, IL-1β, and IL-6) compared to control, while cedrol treatment significantly reduced IL-6 and IL-1β levels (Figures 3H,I). These results suggested that cedrol mitigated DSS-induced inflammation responses by suppressing pro-inflammatory cytokines, highlighting its anti-inflammatory potential in colitis recovery.

### 3.4 Cedrol enhances intestinal barrier function in DSS-colitis mice

To assess the impact of cedrol on intestinal battier recovery, we evaluated the expression of tight junction proteins during the colitis recovery phase using Western blot analysis. As shown in Figures 3K,L, compared to the control group, DSS induction significantly decreased the expression of occludin, claudin-1, and claudin-3. However, cedrol treatment significantly restored these proteins’ expression, indicating that cedrol helped alleviate DSS-induced intestinal barrier damage by upregulating tight junction proteins (occludin, claudin-1, and claudin-3).

### 3.5 Cedrol rephase the intestinal microbiota in DSS-colitis mice

**FIGURE 4:**
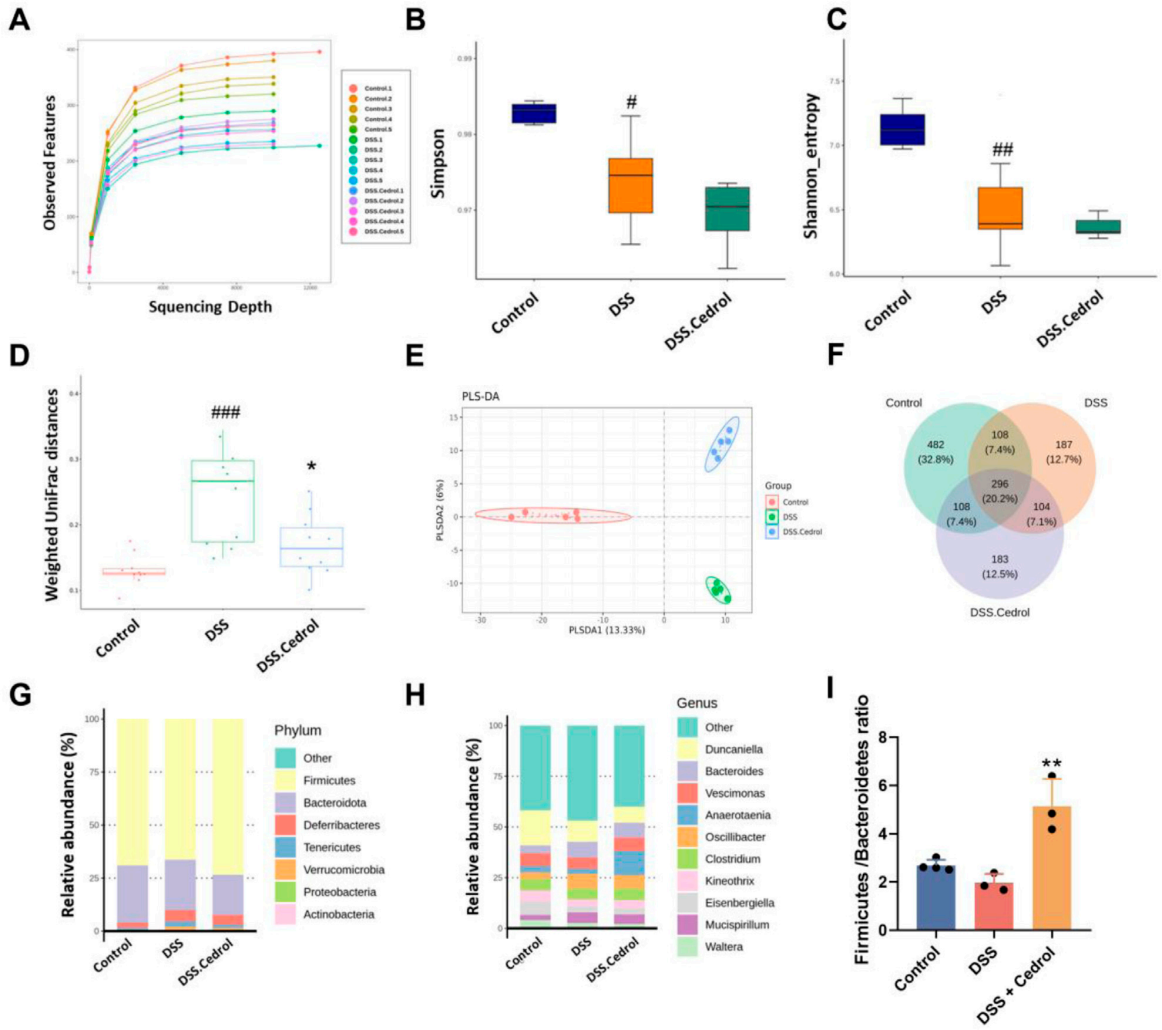
Cedrol altered the diversity and composition of the gut microbiota in colitis mice. **(A)** The rank abundance curve in different groups. **(B)** Simpson index, **(C)** Shannon index, **(D,E)** Principal coordinates analysis (PCoA) of weighted UniFrac distances. Partial Least Squares Discriminant Analysis (PLS-DA) plots were separated by components 1 (13.33%) and 2 (6%) of the total variance from the three groups. **(F)** Venn Diagram. **(G)** Relative abundances of predominant gut microbiota at the phylum level. **(H)** Relative abundances of the gut microbial community at the genus level. **(I)** Firmicutes/Bacteroidota Ratio. The values are expressed as mean ± SD of more than three independent experiments. Statistical differences were analyzed with one-way ANOVA. # vs. control group (^#^ *p* < 0.05, ^##^ *p* < 0.01, ^###^ *p* < 0.001, ^####^ *p* < 0.0001) and * vs. DSS group (*p < 0.05, **p < 0.01). Control, corn oil; DSS, DSS-induced; DSS + Cedrol, DSS induced with cedrol treatment.

The effect of intestinal microbiota treated with cedrol during the colitis recovery phase was evaluated through 16S rRNA gene sequencing. Rarefaction curves confirmed that sequencing depth was sufficient, as all samples reached saturation (Figure 4A). To assess the impact of cedrol on bacterial diversity, we analyzed the gut microbiota’s α-diversity and β-diversity. The α-diversity, reflecting microbial richness, shows a significant reduction in the DSS group via the Shannon and Simpson indices compared to the control group. However, no significant α-diversity was observed between the DSS and cedrol groups (Figures 4B,C). The β-diversity demonstrated distinct microbial compositions among the control, DSS, and cedrol-treated groups. However, cedrol treatment notably restored microbial diversity and can be distinguished from the PLS-DA model (Figures 4D,E). Bacterial composition and abundance analysis at the phylum and genus levels revealed dominant phyla, including Firmicutes, Bacteroidota, Deferribacteres, Tenericutes, and Verrucomicrobia. DSS treatment reduced Firmicutes abundance and decreased the Firmicutes/Bacteroidetes (F/B) ratio, a key indicator of disease. Cedrol treatment significantly restored the F/B ratio (Figures 4F,G,I). Dominant genera included *Duncaniella*, *Bacteroides*, *Vescimonas*, and *Anaerotaenia*. Compared to the control group, *Bacteroides* and *Oscillibacter* abundance increased, while *Duncaniella* abundance decreased in the DSS group. However, cedrol restored microbial abundance (Figure 4H), suggesting that cedrol counters DSS-induced microbial shifts.

**FIGURE 5:**
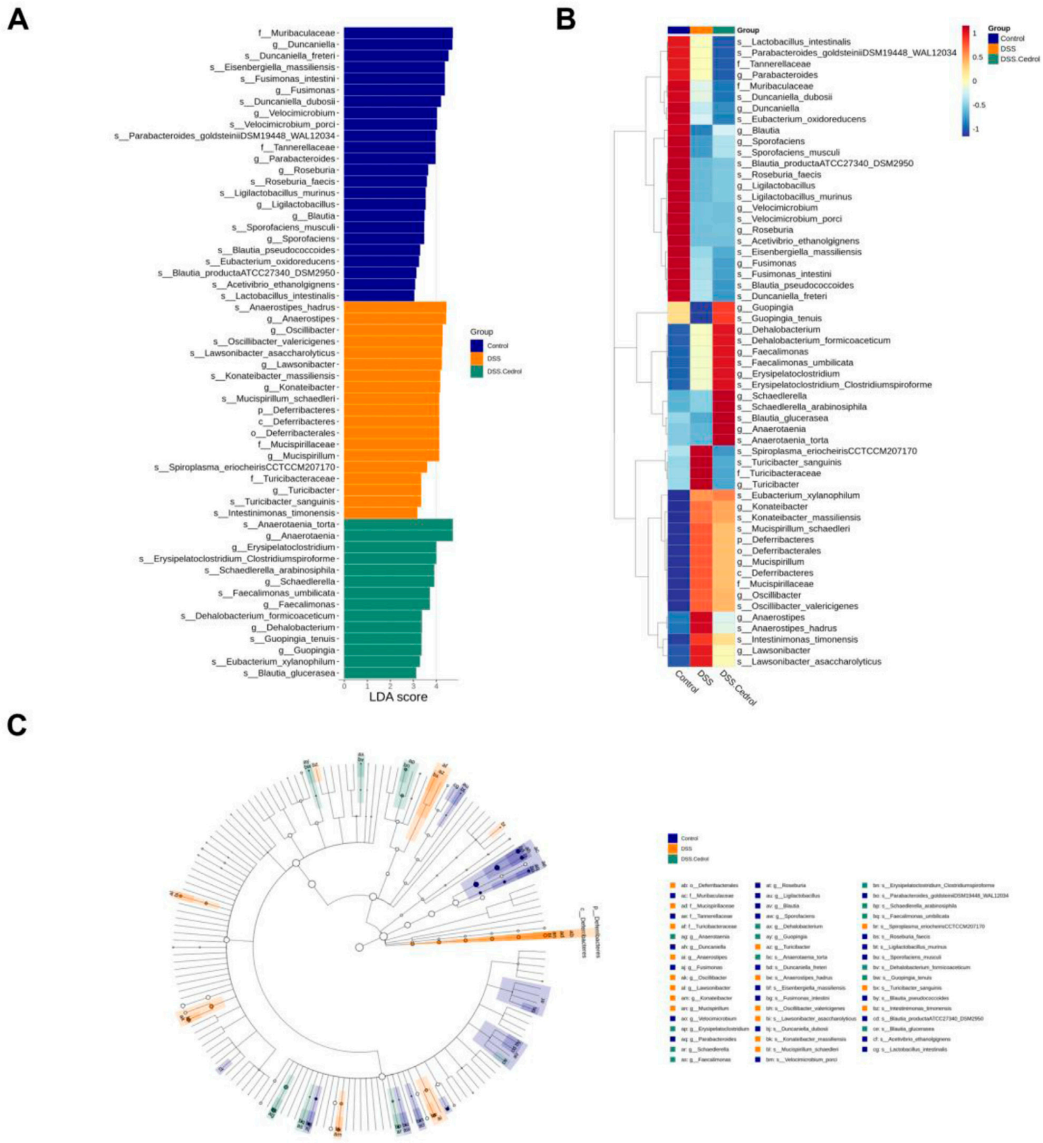
Effects of Cedrol on dominant microorganisms. **(A)** Distribution histogram based on Linear discriminant analysis (LDA), with an LDA score above 3.0. The LDA histogram shows the species with significant differences, which are larger than the preset value. The color of the histogram represents each group, and the influence degree of the species with significant differences between different groups can be expressed according to the length of the histogram. **(B)** Heatmap of the relative abundances from different groups. **(C)** Cladogram based on LDA, with a log LDA score above 3.0. The circles radiating from the inside to the outside represent taxonomic levels from phylum to genus. The diameter of the small circles represents the relative abundance.

LefSe (linear discriminant analysis effect size) identified species with significant abundance differences. With a LDA score threshold >3.0, they were visualized in distribution and cladogram plots (Figures 5A,C). The control group was enriched with *Duncaniella*, *Velocimicrobium*, and *Blautia*, while the DSS group showed higher abundances of *Anaerostipes*, *Lawsonibacter*, and *Turicibacter*. *Anaerotaenia*, *Schaedlerella*, *Faecalimonas*, and *Blautia* were dominant in the cedrol group. The heatmap further illustrated biomarker abundance differences, where *Turicibacter sanguinis* and *Anaerostipes hadrus* were increased in the DSS group compared to controls. Cedrol restored these levels, significantly increasing *Blautia glucerasea* and *Anaerotaenia torta* abundance (Figure 5B).

**FIGURE 6:**
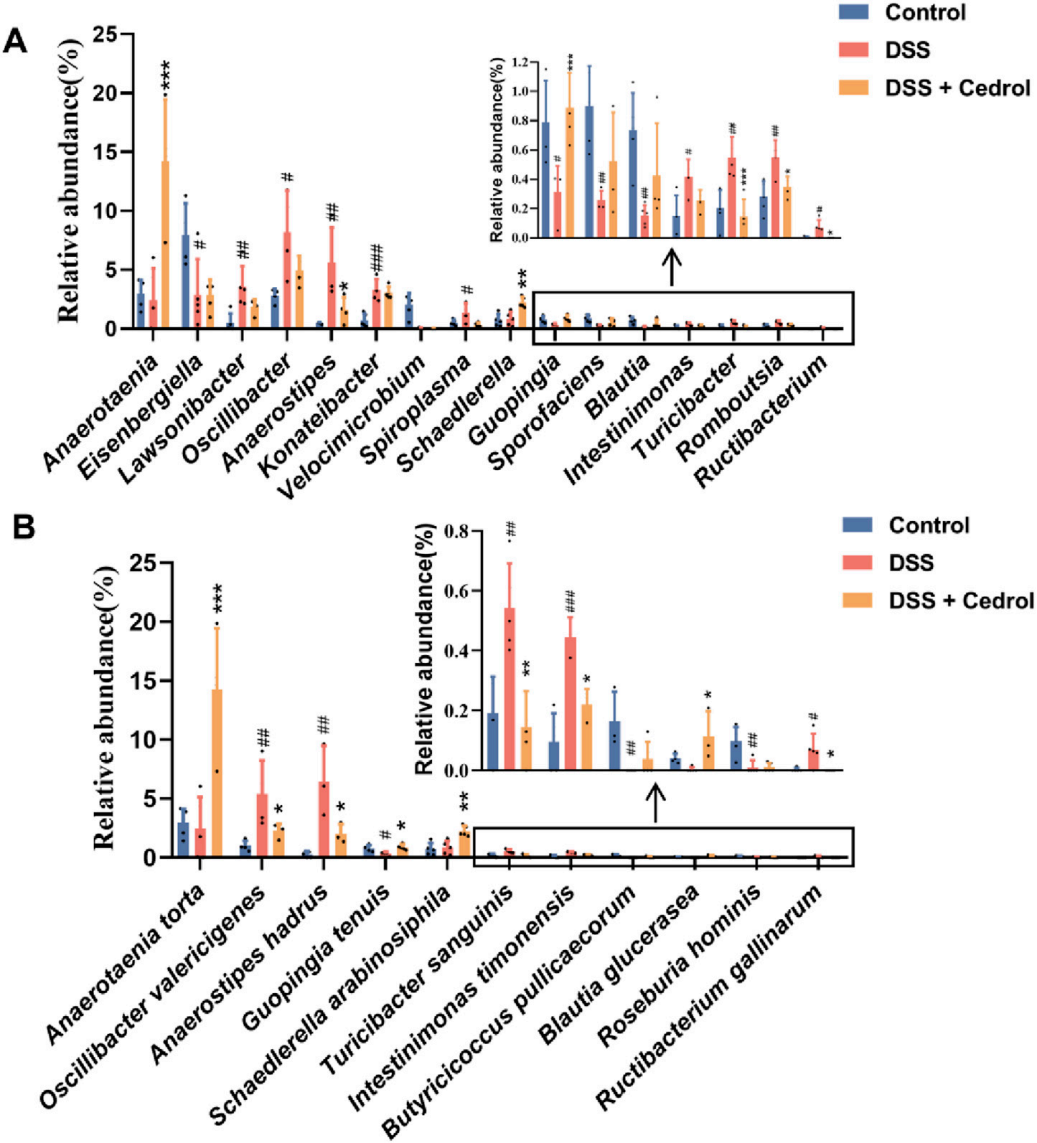
The relative abundance of gut microbes in colitis mice. **(A)** The relative abundance of the genus. **(B)** The relative abundance of species. The values are expressed as mean ± SD of more than three independent experiments. # vs. control group (^#^ *p* < 0.05, ^##^ *p* < 0.01, ^###^ *p* < 0.001) and * vs. DSS group (*p < 0.05, **p < 0.01, ***p < 0.001). Control, corn oil; DSS, DSS-induced; DSS + Cedrol, DSS induced with cedrol treatment.

The microbial abundance of differences among groups was highlighted in Figure 6. The DSS group significantly decreased *Eisenbergiella*, *Velocimicrobium*, *Guopingia*, *Sporofaciens*, and *Blautia* levels, while *Lawsonibacter*, *Oscillibacter*, *Konatebacter*, *Spiroplasma*, *Turicibacter*, *Intestinimonas*, *Romboutsia*, *Ructibacterium*, and *Anaerostipes* were elevated. Cedrol treatment restored the levels of *Anaerostipes*, *Spiroplasma*, *Guopingia*, *Turicibacter*, *Romboutsia*, and *Ructibacterium* (Figure 6A). At the species levels, the DSS group significantly decreased abundances of *Guopingia tenuis*, *Butyricicoccus pullicaecorum*, and *Roseburia hominis*, while *Oscillibacter valericigenes*, *A. hadrus*, *T. sanguinis*, *Intestinimonas timonensis*, and *Ructibacterium gallinarum* were significantly increased. Cedrol treatment restored *O. valericigenes*, *A. hadrus*, *Guopingia tenuis*, *T. sanguinis*, *Intestinimonas timonensis*, and *Ructibacterium gallinarum* abundances (Figure 6B). In summary, DSS induction causes gut microbiota dysbiosis, which cedrol treatment effectively mitigates by restoring microbial diversity and composition.

### 3.6 The levels of short-chain fatty acids (SCFAs)

**FIGURE 7:**
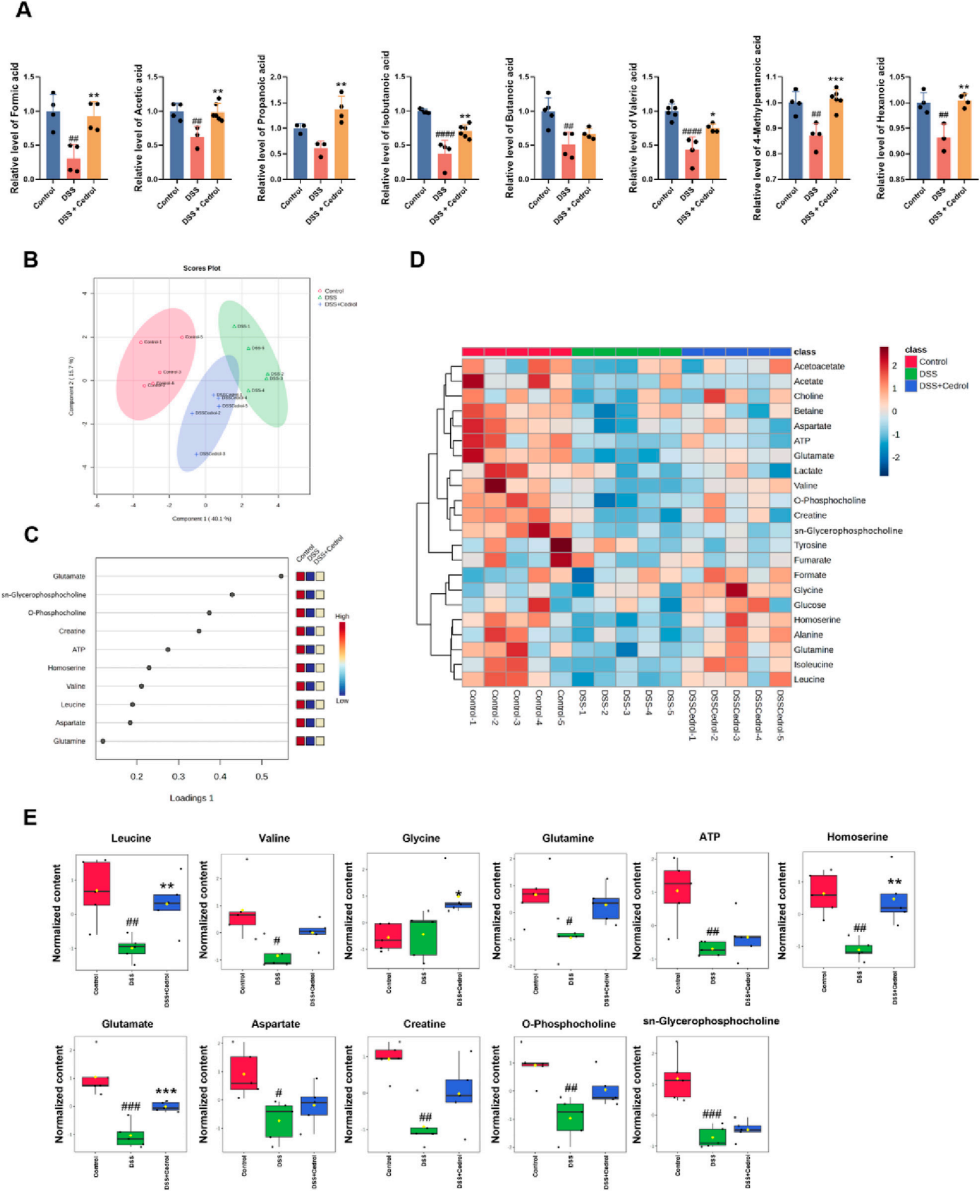
Administration of cedrol improves metabolites in mice feces and colon tissue. **(A)** The fecal concentration of SCFAs was determined using GC-MS. Sparse partial least squares-discriminant analysis (sPLS-DA) analysis of ^1^H NMR spectral data from mice colon tissue. **(B)** The score plots were separated by components 1 (40.1%) and 2 (15.7%) of the total variance in the three groups of mice colon tissue sample extracts. **(C)** The loading plot shows the variables selected by the sPLS-DA model for a given component. **(D)** Heatmap of the metabolic variations from the three groups of mice colon tissue sample extracts. **(E)** The quantitative levels of significant metabolites detected in mice colon tissue extracts. The values are expressed as mean ± SD of more than three independent experiments. Statistical differences were analyzed with one-way ANOVA. # vs. control group (^#^ *p* < 0.05, ^##^ *p* < 0.01, ^###^ *p* < 0.001) and * vs. DSS group (*p < 0.05, **p < 0.01, ***p < 0.001). Control, corn oil; DSS, DSS-induced; DSS + Cedrol, DSS induced with cedrol treatment.

Short-chain fatty acids (SCFAs), produced by the gut microbiota through anaerobic fermentation, are crucial as a main energy source for intestinal cells. In addition to this, SCFAs are involved in regulating cell proliferation, differentiation, and apoptosis, as well as immune responses, energy metabolism, nutrient absorption, and lipid metabolism. In this study, we analyzed SCFA levels using GC-MS and compared them to standards (Supplementary Figure S2; Supplementary Table S1). As shown in Figure 7A, compared to the control group, formic acid, acetic acid, isobutanoic acid, butanoic acid, valeric acid, 4-methylpentanoic acid, and hexanoic acid were significantly reduced in the DSS-induced group. However, cedrol treatment significantly restored formic acid, acetic acid, isobutanoic acid, valeric acid, 4-methylpentanoic acid, and hexanoic acid levels. While propanoic acid levels remained unchanged between the control and DSS groups, cedrol treatment significantly elevated propanoic acid. These finding demonstrates that DSS-induced colitis in mice significantly depletes fecal SCFA, which can be effectively restored through cedrol treatment.

### 3.7 ^1^H NMR analysis of the metabolites in mice colon tissue

Metabolic changes in DSS-induced colitis were analyzed using an NMR spectrometer (Advance III-400, Bruker, United States). Based on previous literature and chemical shift data from HMDB and BMRB, 22 metabolites were identified (Supplementary Table S2).

Metabolomic analysis based on NMR was conducted to assess metabolomic changes following cedrol treatment. Initially, an unsupervised multivariate PCA was applied to examine metabolic differences in colonic tissues. However, the PCA model could not distinctly separate the three metabolite groups (Supplementary Figure S3A). Consequently, supervised analysis methods, including Partial least squares discriminant analysis (PLS-DA) and sPLS-DA, were employed to minimize environmental and systematic errors and achieve more accurate grouping results. Although the PLS-DA model also failed to separate the groups clearly (Supplementary Figure S3B), the sPLS-DA model effectively reduced the dimensionality of the metabolomic data, producing a robust and interpretable model. The performance of the sPLS-DA model was validated through 10-fold cross-validation (CV), assessing model fit and average classification error rates across dimensions. Results indicated that the sPLS-DA model maintained stable estimation error rates, which improved with adequate dimensions (Supplementary Figure S4). This model enabled clear clustering of samples, separating the control, DSS, and DSS + cedrol groups with lower error rates (Figure 7B).

The loadings plot of the sPLS-DA model highlighted metabolites with significant inter-group differences (Figure 7C). Detailed metabolite changes and a heatmap of these variations are shown in Supplementary Figures S5; Figure 7D. Eleven metabolites demonstrate notable differences: leucine, valine, glycine, glutamine, glutamate, aspartate, creatine, *O*-phosphocholine, sn-glycerophosphocholine, ATP, and homoserine (Figure 7E). DSS group exhibited a significant reduction in leucine, valine, glutamine, glutamate, aspartate, creatine, *O*-phosphocholine, sn-glycerophosphocholine, ATP, and homoserine compared to the control group. Cedrol treatment significantly restored the levels of leucine, glutamate, and homoserine, as well as increased levels of valine, glutamine, aspartate, creatine, *O*-phosphocholine, sn-glycerophosphocholine, and ATP. These findings suggested that cedrol treatment may restore DSS-induced metabolic imbalances in the colons of mice.

## 4 Discussion

The pathogenesis of IBD is complex. While current therapeutic strategies effectively alleviate symptoms, they are often limited by poor efficacy and significant side effects. Therefore, the development of more selective, low-side-effect therapies for long-term use is crucial. A key characteristic of IBD is the impairment of the intestinal barrier function. Research suggests that maintaining the integrity of tight junction proteins and promoting intestinal mucosal repair can help slow disease progression (Odenwald and Turner, 2017). Additionally, mitochondrial dysfunction has been identified as an early event in IBD pathogenesis, preceding inflammatory responses and serving as a major trigger for chronic intestinal inflammation (Rath and Haller, 2022). Since the integrity of intestinal epithelial cells relies on energy maintenance, mitochondrial function is crucial for maintaining the intestinal epithelial barrier (Lee et al., 2021). Moreover, imbalances in gut microbiota and their metabolites can disrupt host immunity, epithelial turnover, and overall gut function (Ma et al., 2022). Metabolic processes within intestinal cells are also essential for wound healing, a key factor in effective barrier restoration (Rath and Haller, 2022). Given these interrelated mechanisms, restoring mitochondrial function, rebalancing gut microbiota, and regulating metabolism may offer a promising strategy for repairing the intestinal barrier. In this study, we used an IBD model to investigate cedrol’s protective mechanisms in intestinal disorders. Our findings demonstrated that cedrol alleviates IBD symptoms by preserving intestinal barrier integrity through the restoration of mitochondrial function, gut microbiota balance, and metabolic homeostasis.

Recent research confirms that mitochondrial structural abnormalities, reduced ATP, increased oxidative stress, and decreased mtDNA content are all linked to mitochondrial dysfunction in IBD (Sun et al., 2024). Mitochondrial biogenesis involves mtDNA replication and transcription (Alattar et al., 2022). Previous studies indicated that AMPK was crucial as a regulator of energy metabolism; its loss in intestinal epithelial cells impairs barrier function and disrupts tight junction integrity (Sun et al., 2017). Conversely, SIRT-1 overexpression significantly enhances ATP production and reduces mitochondrial damage (Huang et al., 2021). Our study found that cedrol upregulates SIRT-1, PGC-1α, Nrf2, *p*-AMPK/AMPK, and TFAM protein expression, thereby restoring mitochondrial biogenesis impaired by DSS induction. Additionally, cedrol increased TOMM20 protein expression on the mitochondrial membrane and significantly upregulated mtDNA mRNA expression, promoting ATP generation and reducing DSS-induced mitochondrial damage.

The IBD critical symptom is the disruption of the intestinal barrier function, primarily regulated by tight junction proteins in intestinal cells, which prevent the translocation of luminal bacteria and toxins. Research has established that the function of this epithelial barrier is closely associated with gastrointestinal diseases, with tight junction integrity being essential to slowing disease progression (Odenwald and Turner, 2017). The maintenance of tight junctions in intestinal epithelial cells is energy-dependent, and ATP depletion has been shown to promote compromises in epithelial tight junction integrity (Lee et al., 2021). Additionally, studies indicate that mitochondrial function within intestinal epithelial cells is remodeled during recovery from acute colitis, with mitochondrial biosynthesis aiding the restoration of the colonic mucosa’s structure and function (Lan et al., 2023). Our research demonstrates a significant decrease in mitochondrial biogenesis proteins and mitochondrial function, as evidenced by reduced mtDNA and ATP content in DSS-induced colitis mice. However, cedrol treatment mitigates mitochondrial damage. During the recovery phase, cedrol increases tight junction protein expression compared to DSS-treated control. We hypothesize that DSS-induced mitochondrial biogenesis impairment reduces ATP production, limiting the energy supply required for intestinal barrier repair. Cedrol appears to restore ATP levels, supporting the repair of tight junction proteins during colitis recovery.

Besides impairing the intestinal barrier, IBD disrupts gut microbiota composition, and restoring this balance can effectively alleviate IBD symptoms. DSS treatment has been reported to affect fecal DNA amplification, often resulting in unsuccessful 16S rRNA gene sequencing from samples collected during the acute phase (Kerr et al., 2011), which was also observed in our study. To accurately capture microbiota dynamics following acute inflammation, we therefore performed gut microbiota sequencing during the recovery phase. Consistent with previous reports, a decrease in the abundance of Firmicutes was observed (Matsuoka and Kanai, 2015). In this study, DSS-induced mice exhibited a reduction in beta diversity, indicating a decline in species diversity (Figures 4C,D). The DSS-induced group also reduced the abundance of Firmicutes, which is consistent with prior findings. Another critical target in this context is the Firmicutes/Bacteroidetes (F/B) ratio, which relates to various diseases since Firmicutes and Bacteroidetes dominate the gut microbiota. Previous literature reports that the F/B ratio is lower in DSS-induced mice (Mo et al., 2022). Our results show that cedrol treatment restored Firmicutes abundance, reshaped microbiota composition by increasing microbial diversity, and restored the F/B ratio (Figure 4H). *Blautia* is an anaerobic bacterium at the genus level with probiotic properties linked to host health regulation and metabolism. Notably, *B. glucerasea* has shown antibacterial properties and is effective against pathogens linked to inflammatory and metabolic disease (Mao et al., 2024). Our results indicate that DSS induction significantly reduces *Blautia* abundance compared to the control, while cedrol treatment restores *Blautia* and *B. glucerasea* abundance. Previous studies suggest that *A. hadrus* BPB5 exacerbates DSS-induced colitis (Zhang et al., 2016). In our experience, we observed *Anaerostipes* and *A. hadrus* significantly increased in DSS-induced colitis, suggesting a potential role in colitis pathogenesis; however, cedrol treatment alleviated this increase. Additionally, *Turicibacter* is associated with host inflammation and influences bile acid and lipid metabolism (Lynch et al., 2023). Furthermore, *Anaerotaenia torta*, a newly identified species within the *Clostridium* cluster XIVa, is a symbiotic gut bacterium beneficial for intestinal balance (Ueki et al., 2016). *Clostridium* has been shown to alleviate inflammation and produce butyrate, which strengthens the intestinal barrier (Guo et al., 2020). In our study, *Anaerotaenia torta* significantly increased in the cedrol treatment group, suggesting it may aid in protecting the intestinal barrier and alleviating colitis. In summary, 16S rDNA sequencing analysis of the gut microbiota reveals that cedrol treatment mitigates microbial imbalance induced by colitis, enhancing gut microbiota balance and potentially supporting colitis recovery.

SCFAs, which are terminal products of bacterial fermentation in the gut, play a crucial role as energy sources not only for the gut microbiota but also for the host’s intestinal epithelial cells (IECs). They play key roles in maintaining intestinal homeostasis, modulating host immune responses, and inhibiting pathogens' growth, thus reducing inflammation. This highlights the critical role of gut microbiota and their metabolites in preserving intestinal barrier integrity (Ma et al., 2022). For instance, formic acid, acetate, propionate, and butyrate have been shown to exhibit antimicrobial effects and upregulate tight junction protein expression, thereby promoting intestinal function and barrier repair (Ma et al., 2012; Tong et al., 2016; Luise et al., 2017; Yue et al., 2022). Prior research has confirmed that certain SCFA levels decreased in DSS-induced colitis models, with acetic acid, propionic acid, isobutyric acid, and butyric acid significantly reduced in the DSS group compared to the controls (Li et al., 2023). In alignment with these findings, our study revealed that cedrol treatment restored these SCFA levels, indicating that cedrol mitigates DSS-induced colitis by increasing SCFA levels, thereby enhancing tight junction protein expression and strengthening barrier function.

Previous studies have shown that IBD disrupts the body’s metabolic processes. For example, Dong et al. (2013) employed NMR analysis to investigate metabolic changes in DSS-induced colitis mice, observing reduced levels of phosphocholine, glycerophosphocholine, and taurine (Dong et al., 2013). Phosphocholine and glycerophosphocholine are metabolites in the choline synthesis pathway, with choline being a main component in the formation of membrane phospholipids. A deficiency in choline can impact gene expression related to cell proliferation, differentiation, and apoptosis (Michel et al., 2006). In addition, DSS-induced colitis has been linked to amino acid imbalances (Hong et al., 2010). Our research showed significantly reduced levels of amino acids in DSS-induced colitis mice, consistent with previous studies. Amino acids play a key role in intestinal growth and maintain barrier function. Specifically, glutamine, glutamate, and aspartate serve as primary fuels for the small intestinal mucosa, supporting ATP metabolism in the intestine (Wu, 1998). Amino acids also aid in synthesizing proteins and peptides critical for normal intestinal structure and function, as well as sustaining energy for intestinal bacteria growth (Dai et al., 2015). Studies indicate that increasing amino acid levels can promote mucin production, support microbial flora, and enhance the protection and repair of intestinal epithelial tissue (Faure et al., 2006). Furthermore, our study found significantly lower ATP levels in the colons of DSS-induced colitis mice compared to controls, affirming the energy demands of the intestinal epithelium for stability. Cedrol treatment was able to restore phospholipids, amino acids, and ATP metabolism in the colons, supporting the repair and maintenance of the intestinal epithelial damage caused by DSS induction. During the recovery phase, cedrol promoted epithelial repair and facilitated the recovery from colitis.

The pathophysiology of IBD involves a tightly interconnected axis of intestinal microbiota, epithelial metabolism, mitochondrial function, and barrier integrity. Gut microbiota-derived metabolites, such as short-chain fatty acids (SCFAs), support mitochondrial biogenesis and energy production in intestinal epithelial cells, promoting epithelial differentiation and barrier maintenance (Tan et al., 2014; Park et al., 2016). Conversely, inflammation and dysbiosis impair mitochondrial function, leading to ATP depletion, reactive oxygen species accumulation, and compromised epithelial regeneration, which further destabilizes the gut barrier (He et al., 2002). Intestinal epithelial metabolism also feeds back to shape microbiota composition, with metabolic shifts during inflammation promoting the growth of facultative anaerobes and further exacerbating dysbiosis (Litvak et al., 2018). Thus, intestinal barrier integrity, epithelial metabolism, mitochondrial function, and gut microbiota form a tightly interconnected axis that drives IBD pathophysiology, highlighting potential therapeutic targets for IBD.

Building on this interconnected axis, mitochondrial dysfunction and ATP depletion are central to IBD pathogenesis. Our study confirmed that DSS-induced colitis reduced ATP levels in mouse colons, while cedrol effectively restored these levels, supporting its protective effects on mitochondrial function. From a traditional Chinese medicine (TCM) perspective, these findings align with the concept of spleen Qi deficiency, characterized by impaired energy metabolism and gastrointestinal vulnerability. Qi deficiency has been linked to mitochondrial dysfunction, reduced oxidative phosphorylation, and impaired intestinal barrier integrity (Roediger, 1980; Li et al., 2015; Denson, 2020; Luo et al., 2022). Interventions that enhance mitochondrial biogenesis and ATP synthesis, such as cedrol, may therefore alleviate both the biochemical and TCM-defined deficits, restoring intestinal energy balance and promoting mucosal healing. This integrative view highlights how modulating mitochondrial function can serve as a therapeutic strategy in IBD while providing a scientific basis for the TCM notion of Qi replenishment.

## 5 Conclusion

In conclusion, cedrol exerts its protective effects by promoting mitochondrial biogenesis, resulting in increased mtDNA mRNA expression and enhanced ATP production. This enhanced energy supply supports the expression of tight junction proteins, facilitating intestinal barrier repair. Additionally, cedrol helps restore the balance of gut microbiota, short-chain fatty acids (SCFAs), and metabolites, thereby alleviating symptoms of colitis. This study is the first to examine an IBD model through the integrated lens of mitochondrial function enhancement, gut microbiota stability, and metabolic regulation. By evaluating both the induction and recovery phases of colitis, our findings demonstrate that cedrol not only attenuates acute inflammatory injury but also promotes sustained mucosal healing during remission. This dual-phase assessment underscores the clinical relevance of cedrol’s therapeutic potential, highlighting the interplay between mitochondria, gut microbiota, metabolism, and intestinal barrier integrity, and offering a novel alternative to traditional anti-inflammatory strategies in IBD treatment.

## Data Availability

The datasets presented in this article are not readily available because No. Requests to access the datasets should be directed to Sheng Yang Wang taiwanfir@dragon.nchu.edu.tw.

## Abbreviations

Inflammatory bowel disease (IBD); Mitochondrial biogenesis (MB); Tight junction (TJ); Short-chain fatty acid (SCFA); Dextran sulfate sodium (DSS); Disease activity index (DAI)
