# Supplementary material for: Cedrol ameliorates inflammatory bowel disease via mitochondrial biogenesis, gut microbiota restoration, and intestinal barrier repair
Source: Front Pharmacol. 2026 Jan 2;16:1619537. doi: 10.3389/fphar.2025.1619537 (PMC12808353; doi:10.3389/fphar.2025.1619537)
Supplement: Supplementary file 1 [file Supplementaryfile1.docx]

**Supplementary Material**

| 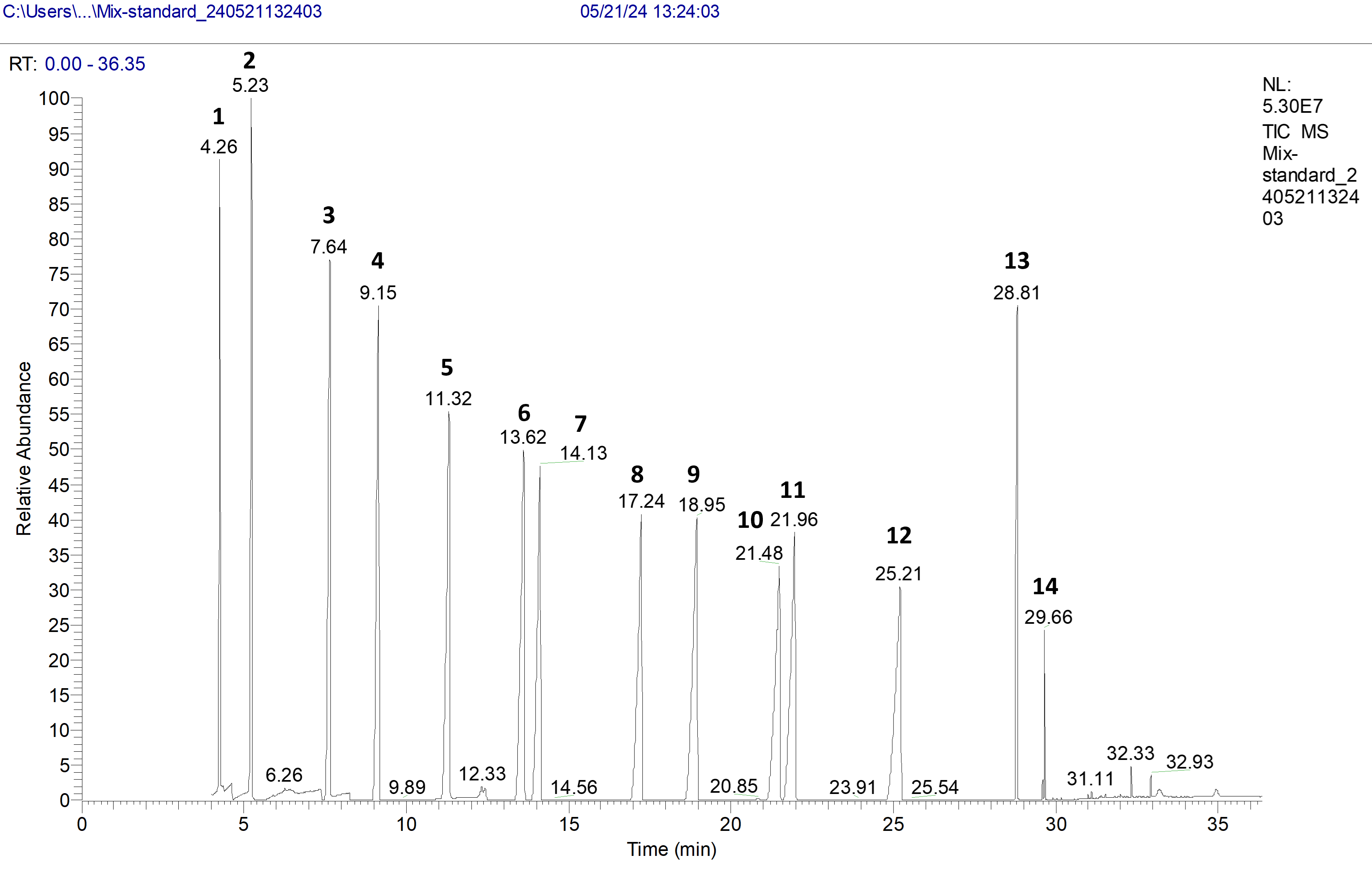 |
| --- |
| **Fig. S1** The chromatogram of standard samples was determined using GC-MS.  SCFAs:1, Formic acid; 2, Acetic acid; 3, Propanoic acid; 4, Isobutanoic acid; 5, Butanoic acid; 6, 2-Methylbutanoic acid; 7, Isovaleric acid; 8, Valeric acid; 9, 2-Ethylbutanoic acid; 10, 3-Methylpentanoic acid; 11, 4-Methylpentanoic acid; 12, Hexanoic acid; 13, Heptanoic acid; 14, Lactic acid. |


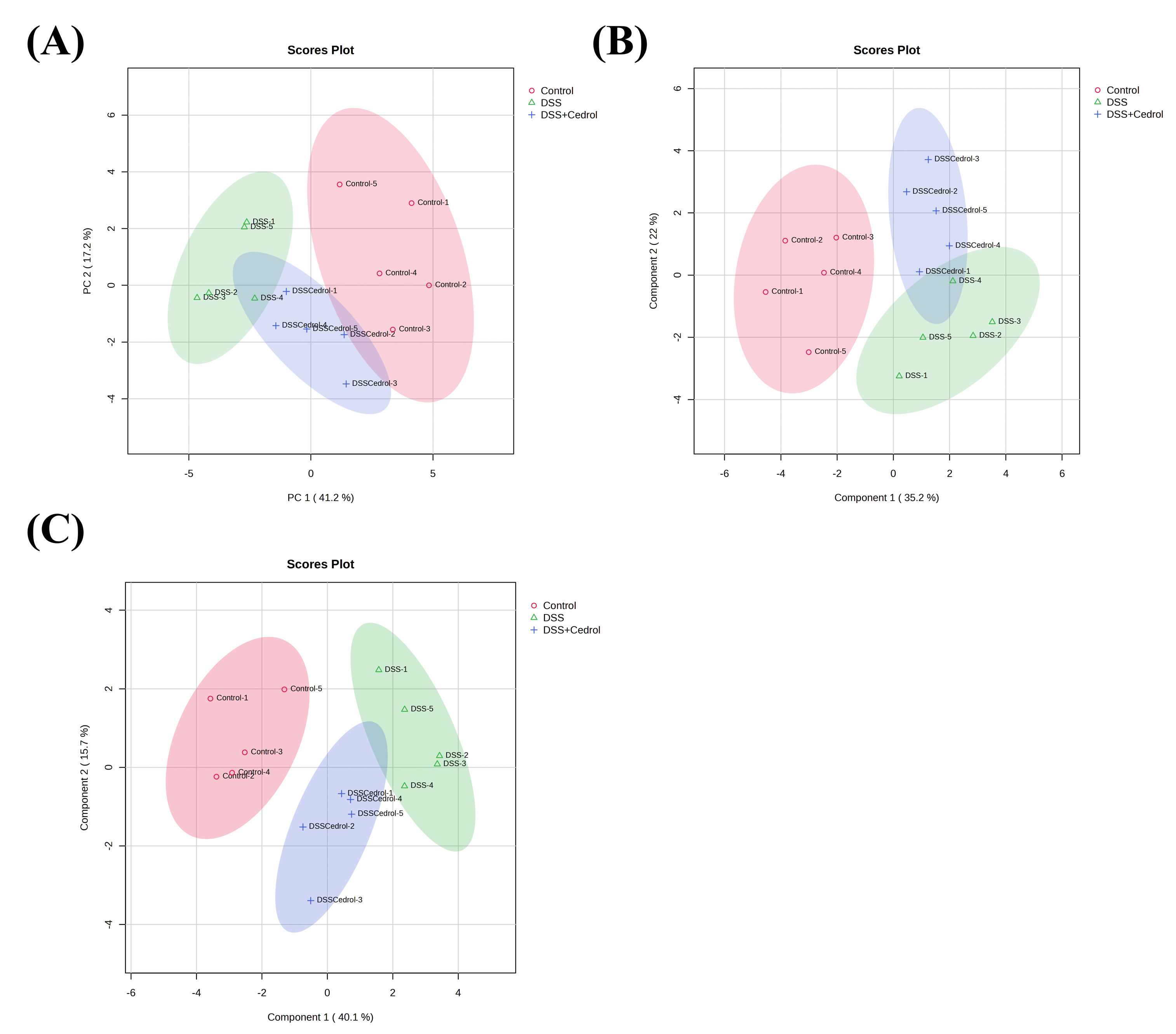


**Fig. S2** The scatter plots of ^1^H NMR spectral data from the three groups of mice colon tissue sample extracts.

(A) Principal components analysis and (B) Partial least squares-discriminant analysis (PLS-DA) obtained for ^1^H NMR spectra of mice colon tissues.

| 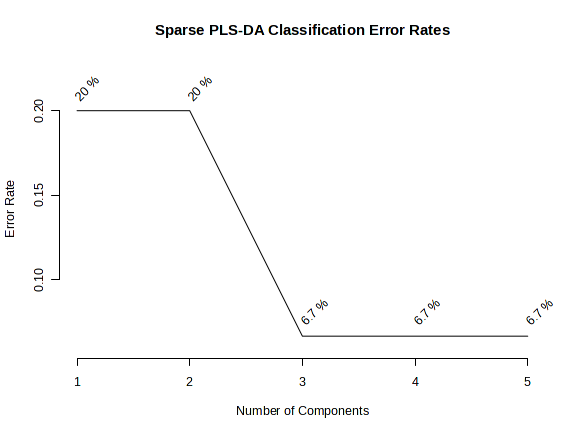 |
| --- |
| **Fig. S3** The error rates of sPLS-DA classification. |

| 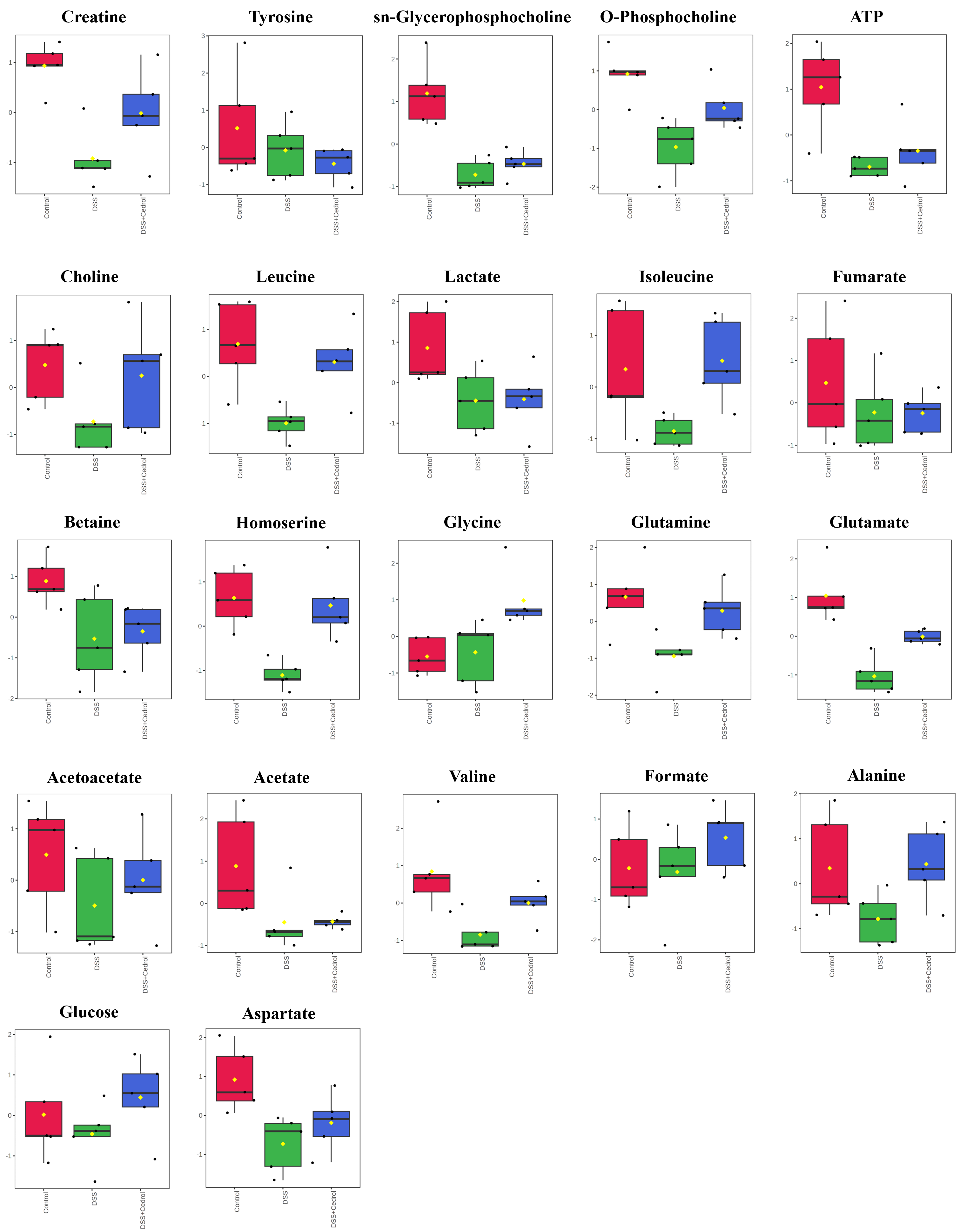 |
| --- |
| **Fig. S4** Quantitative levels of corresponding metabolites detected in mice colon tissue extracts. |

**Table. S1** GC-MS identified the retention time of standard metabolites (SFTA) in feces.

| **NO.** | **Retention time (min)** | **Metabolite Name** | **Identification** |
| --- | --- | --- | --- |
| 1 | 4.26 | Formic acid | MS/KI/ST |
| 2 | 5.23 | Acetic acid | MS/KI/ST |
| 3 | 7.64 | Propanoic acid | MS/KI/ST |
| 4 | 9.15 | Isobutanoic acid | MS/KI/ST |
| 5 | 11.32 | Butanoic acid | MS/KI/ST |
| 6 | 13.62 | 2-Methylbutanoic acid | MS/KI/ST |
| 7 | 14.13 | Isovaleric acid | MS/KI/ST |
| 8 | 17.24 | Valeric acid | MS/KI/ST |
| 9 | 18.95 | 2-Ethylbutanoic acid | MS/KI/ST |
| 10 | 21.48 | 3-Methylpentanoic acid | MS/KI/ST |
| 11 | 21.96 | 4-Methylpentanoic acid | MS/KI/ST |
| 12 | 25.21 | Hexanoic acid | MS/KI/ST |
| 13 | 28.81 | Heptanoic acid | MS/KI/ST |
| 14 | 29.66 | Lactic acid | MS/KI/ST |

MS, NIST library and literature; KI, Kovats index; ST, authentic standard compounds. Kovats index on DB-5MS column in reference to *n*-alkanes.

**Table. S2** The chemical shift of different metabolites in mice colon tissue extracts was identified by ^1^H-NMR.

| **NO.** | **Metabolite Name** | **Chemical shift (ppm)** | **Reference** |
| --- | --- | --- | --- |
| 1 | Leucine | 0.98(t) | [1,2] |
| 2 | Isoleucine | 0.95(t), 1.02(d), 3.6(d) | [1,2] |
| 3 | Valine | 1.0(d), 1.06(d) | [1,2] |
| 4 | Lactate | 1.33(d), 4.05(t) | [1,2] |
| 5 | Alanine | 1.48(d) | [1,2] |
| 6 | Acetate | 1.90(s) | [1,2] |
| 7 | Homoserine | 2.08(m) | HMDB0000719 |
| 8 | Acetoacetate | 2.22(s) | [3] |
| 9 | Glutamine | 2.13(m), 2.45(m), 3.71(t) | [1,2] |
| 10 | Glutamate | 2.04(m), 2.11(m),  2.54(m), 3.72(dd) | [1,2] |
| 11 | Aspartate | 2.84(m), 2.99(m), 3.97(m) | [1] |
| 12 | Creatine | 3.04(s) | [1,2] |
| 13 | Choline | 3.21(s) | [1,2] |
| 14 | O-Phosphocholine | 3.23(s) | [2] |
| 15 | sn-Glycerophosphocholine | 3.24(s) | [1] |
| 16 | Betaine | 3.26(s) | [1,2] |
| 17 | Glucose | 3.31-3.84(m), 4.58(d), 5.18(d) | [1,2] |
| 18 | Glycine | 3.50(s) | [1,2] |
| 19 | Fumarate | 6.52(s) | [2] |
| 20 | Tyrosine | 6.85(d), 7.18(d) | [1,2] |
| 21 | ATP | 8.24(s), 8.51(s) | [1,2] |
| 22 | Formate | 8.44(s) | [1,2] |

**Reference**

1. Kostidis S, Addie RD, Morreau H, Mayboroda OA, Giera M. Quantitative NMR analysis of intra- and extracellular metabolism of mammalian cells: A tutorial. Analytica Chimica Acta. 2017; 980: 1-24.

2. Carneiro TJ, Araújo R, Vojtek M, Gonçalves-Monteiro S, Diniz C, Batista de Carvalho ALM, et al. Multi-Organ NMR Metabolomics to Assess In Vivo Overall Metabolic Impact of Cisplatin in Mice. Metabolites. 2019; 9(11): 279.

3. Zira AN, Theocharis SE, Mitropoulos D, Migdalis V, Mikros E. 1H NMR Metabonomic Analysis in Renal Cell Carcinoma: a Possible Diagnostic Tool. Journal of Proteome Research. 2010; 9(8): 4038-4044.
